# Meanings of feeling well among women with Parkinson's disease

**DOI:** 10.3402/qhw.v10.28730

**Published:** 2015-10-20

**Authors:** Malin Olsson, Carina Nilsson

**Affiliations:** Division of Nursing, Department of Health Science, Luleå University of Technology, Luleå, Sweden

**Keywords:** Interviews, lived experience, Parkinsons disease, nursing, well-being, women

## Abstract

We conducted a qualitative inquiry to describe the meanings of feeling well as experienced by women with Parkinson's disease. Nine women were interviewed and we analysed the interviews using a reflective lifeworld approach based on phenomenological epistemology. We present the analysis as five constituents: the body as unnoticed; being able to move on; feeling joy by being connected; finding peace and harmony; and being the director of one's own life. Our findings can be used to understand and promote well-being among women with Parkinson's disease. In care meetings, knowledge about the lived and experienced health processes supports the women's striving to not let illness dominate their experience of daily life.

In this study, we report the findings of a qualitative inquiry. We interviewed nine women with Parkinson's disease (PD) about their experiences of feeling well. The interviews were analysed using the reflective lifeworld research (RLR) approach, based on phenomenological epistemology as described by Dahlberg, Dahlberg, and Nyström (2008).

Living with long-term illness has been studied predominantly with a focus on the obstacles that being ill causes in the lives of people affected (cf. Bury, 1982; Charmaz, 1987, 2006; Hurd Clarke & Griffin, 2008; Morse, 1997; Thorne & Paterson, 1998). Today, it is relatively well known that bodily disruptions can impose on and alter the everyday lives of persons living with long-term illnesses (cf. Carrel, 2008; Olsson, Lexell, & Söderberg, 2008; Toombs, 1993). According to Carrel (2008), the experience of bodily disruption, as related to being ill, consequently gives rise to feelings of uncertainty in one's everyday life. Among women with long-term illnesses, Kralik, Brown, and Koch (2001) found that illness was perceived as an intruder that disturbs the perception of the self.

PD is one of the most common neurodegenerative disorders. It is a chronic and progressive disease that strongly affects the daily lives of patients (Gaig & Tolosa, 2009). The aetiology is complex, and the four cardinal symptoms are tremors, bradykinesia, rigidity, and postural instability (Albin, 2006; Backer, 2006; Gaig & Tolosa, 2009). Other manifestations include various non-motor symptoms such as autonomic dysfunction, cognitive changes, and sleep disturbances (Gaig & Tolosa, 2009). Braak et al. (2002) showed that the onset of the particular disease process begins several years earlier than the appearance of symptoms. The manifestations of PD usually start to appear between 40 and 50 years of age, but this can vary.

Studies (Haahr, Kirkevold, Hall, & Ostergaard, 2010; Olsson, Stafström, & Söderberg, 2013; Rahman, Griffin, Quinn, & Jahanshahi, 2008) have demonstrated that living with PD implies a daily life where the unfamiliar body and the experience of being fatigued restrain the ability to engage in habitual routines. Because the body is setting the agenda and due to a lack of energy, people are forced to structure their daily lives in ways that help them to manage the loss of energy they experience. Distinct also is the struggle against the uncertainty that comes with the illness's unpredictable character (Bramley & Eatough, 2005; Olsson et al., 2013; Sunvisson & Ekman, 2001; Sunvisson, Habermann, Weiss, & Benner, 2009).

Focusing on health-related quality of life, studies (Busenbark, Nash, Nash, Hubble, & Koller, 1991; Longstreth, Nelson, Linde, & Munoz, 1992) have shown that the effects of PD have many negative impacts on the quality of life of those affected. The difference between people with PD and healthy matched controls has also been shown to be significant (Busenbark, et al., 1991; Longstreth et al., 1992). More recent studies (Schrag, 2006; Schrag, Jahanshahi, & Quinn, 2000a, 2000b) have pointed out the importance of the subjective evaluation of quality of life in the assessment and treatment of patients diagnosed with PD. Longitudinally, Karlsen, Tandberg, Årsland, and Larsen (2000) showed that, despite treatment, there is significant deterioration concerning health-related quality of life among persons with PD, a deterioration that concerns not only physical mobility, but also emotional reactions and social isolation. It has also been demonstrated that the quality of life among people with PD is clearly affected by motor as well as non-motor symptoms (Phu et al., 2014; Qin et al., 2009; Santos-Garcia & De la Fuente-Fernandez, 2013). Studies (Fleming, Tolson, & Schartau, 2004; Schartau, 2003) with a specific focus on middle-aged women with PD revealed experiences of major changes in everyday living, and, in general, the women's main concerns were seen as related to womanhood, and more specifically, the ability to live up to contemporary images of womanly skills and appearance. Despite the well-known constraints, uncertainties, and bodily hindrances reported among people with PD, Olsson et al. (2013) have discussed how women with PD strived to become accustomed to their unfamiliar bodies and that they described days when they had the strength to accomplish what they wanted to do.

In summary, it can be said that the body of literature shows that research concerning well-being and health related to PD has taken mainly a quantitative approach. It is also clear that the main focus lies within the problems of daily living that persons who live with PD are faced with. There is a lack of studies that focus solely on women's conditions from a lifeworld approach. The literature review not only shows the important focus on understanding specific symptoms, but it also reveals the gap in knowledge concerning what it means for women with PD to feel well in their daily lives. As a result, a lifeworld-led approach is overlooked, and this might in effect limit the possibilities of understanding and also formulating adequate support for women who strive to feel well despite living with PD. Thus, the aim of this study is to describe meanings of feeling well among women with PD.

## Method

In this study, a phenomenological approach has provided us with guidelines and practices for gathering data and conducting research. The starting point builds upon a focus on the lifeworld with the assumption that attention is paid to the subjective body. The lifeworld theory is here an epistemological and methodological tool with which the everyday lives of women living with PD can be understood (cf. Dahlberg, Dahlberg, & Nyström, 2008). Well-being or the pursuance of life projects can, according to Dahlberg and Segesten (2010), be seen as a phenomenon that needs to be described and problematized when the goal is to support people in their health processes. The support of health and well-being often assumes an access to people's lifeworlds and their natural context. Starting with Merleau-Ponty (1996), personal experience or perception is always incorporated in the lived body. When one is ill or is hindered from engaging in valued activities of everyday life, one's perception of the surrounding world becomes altered as the environment is perceived differently (Leder, 1990; Olsson et al., 2008; Toombs, 1993). The lived engagement with the environment can thereby be seen as disrupted, a matter that can be understood as distinct from the experience of feeling well and unconstrained (cf. Gadamer, 1996). In this study, the phenomenological approach directs our attention to the lived world and the lived body. The chosen approach provides us with tools for gaining a phenomenological attitude when conducting this study (cf. Dahlberg et al., 2008).

### Participants and procedure

In the study, nine women diagnosed with PD participated. The criteria for participation were that they were adult women diagnosed with PD, and that their daily lives were affected by their illness. Their ages ranged from 46 to 65, and as a group they had experienced symptoms for 3–19 years; five women were married, two were cohabiting, and two were widowed; five were receiving a disability pension, one a state pension, and three were working part time. The women's participation was arranged through a support group and a patient association in northern Sweden. The women were contacted by means of chain referral and previously chosen informants in the support group, and the patient association contacted the women about the study. All of those contacted were interested in participating. Letters were sent to the women to invite them to participate, give information, and obtain informed consent. After they had agreed to further contact, the first author (MO) telephoned each woman to arrange an interview.

### Lifeworld interviews

Personal audiotaped interviews were conducted using a reflective lifeworld approach (cf. Dahlberg et al., 2008). All of the women preferred to be interviewed in their homes, and this provided a “here and now” atmosphere to the situation. The women were asked to talk about their lived experiences of feeling well and the first author (MO) strived for immediacy and presence. Probing questions such as “Can you tell me more?”, “What is it like?”, and “Can you give me an example?” were engagingly asked to clarify their experiences. In order to avoid social jargon, the first author (MO) encouraged the interviewees to reflect on the phenomenon. This was done by constantly questioning and responding to the narrations of the interviewees. The use of directing and follow-up questions was used to maintain initiative and control during the interviews. The interviews lasted between 40 and 60 min and were transcribed as text. Data collection was performed during 2013 by the first author (MO). The interviews gave varied descriptions of the studied phenomenon, and they did not contain contradicting expressions.

### Ethical considerations

All of the women gave their informed consent both verbally and in writing. The participants were guaranteed confidentiality and an anonymous presentation of the findings. According to Swedish law (Swedish Health Care Act, 2003, p. 460), an ethical review was not necessary, but informed consent was obtained and confidentiality assured (World Medical Association Declaration of Helsinki, 2008).

### Data analysis by reflective lifeworld research

Analysing and describing phenomena and their essences are methodological goals in RLR, where a structure of essential meanings that explicates a phenomenon is put forward. The comprehension of essences is to be understood as grounded in the everyday experience of the world, and lifeworld descriptions represent the focus of attention (Dahlberg, 2006). The conduct of RLR analysis has a tripartite structure and is to be understood as a movement between the whole to its parts to the whole, which is significant when analysing text for meanings (Dahlberg et al., 2008). When analysing, the knowledge of the whole generates questions about the parts, and knowledge about the parts generates new questions about the whole (Bengtsson, 1991; Dahlberg et al., 2008).

In this study, the transcribed interviews were listened to, read, and reread to gain insight into the text as a whole. After gaining insight into the text as a whole, both authors focused their reading on exploring the phenomenon and searching for meanings of the text in its parts. Meanings of the parts were then related to each other by similarities, as a structure of meaning was explored by clustering meaning-carrying parts. During the clustering, the parts of the text were clustered into groups of meaning, and several clusters could be unified by meaning. In the process of clustering, we abstracted the meanings in order to reach the phase of essence formation. Finally, we viewed the material as a whole, and patterns of meanings were described by the formation of essence. When conducting the analysis, both authors explored patterns of meanings from the interview text, and an invariant essence of the phenomenon was explicated. The essence was evident in all clusters, and in all of the narrations that we analysed. In the essence, we synthetized the phenomenon's structure of meaning and we have been using citations for contextualization and concretization. According to Dahlberg et al. (2008), exploring meanings in data implies a phenomenological attitude where the researcher's preunderstanding is bridled; this implies a scrutinizing stance in relation to the meanings of the studied phenomenon; and reflecting on the whole as meanings come to light is a necessity (Dahlberg, 2006). In a phenomenological attitude, our presupposed approach to the world was bridled in favour of not making definite what is indefinite, and instead questioning our own judgements of a general considered certainty (cf. Dahlberg & Dahlberg, 2003). In this analysis, we continuously identified specific meanings in relation to the whole of the text, in a process of moving back and forth between the particular and the background of the entire material.

## Findings

The essence of meanings of feeling well, as experienced by women with PD, seemed to encompass a situation where life feels pleasant and where it is possible to find repose in self-awareness and not feeling estranged. Feeling well is understood in relation to living with a long-term illness, and it encompasses a stance where illness is not the dominant experience in daily living. When unfamiliar bodily sensations are milder and when it is possible to move away from the burdensome body, it is also possible to feel well despite living with PD. Due to finding the body as background or forgotten, it is possible to focus on the surrounding environment, and to be directed towards charitable life events. Meanings of feeling well imply a movement between being active and being in stillness. We present the analysis in five constituents: *The body as unnoticed*, *Being able to move on*, *Feeling joy by being connected*, *Finding peace and harmony*, and *Being the director of one's own life*. The constituents are presented in the following sections and illustrated with quotations from the interviews.

### The body as unnoticed

The experience of feeling well entails a notion of the body as no longer being the focus in the everyday lives of women with PD. The women described that they felt well when the body was pain free and less constrained by tremor or over mobility. Listening to music was said to be relaxing; and the women described the perceptions of the body as agile and functioning smoothly. They expressed that their bodies were working in a familiar manner, and they contrasted experiences of being ill and being healthy. They said that they remembered how their bodies used to work before their illness; when their bodies felt agile and they were able to move around effortlessly, they also felt well despite being ill. The women described their desire to be able to do things as they had before they became ill and wanting to retain as many abilities as possible. They said that they felt well when the body felt normal and when it felt easy to walk. For women with PD, feeling well encompassed being able to do ordinary things, for example, meeting friends, shopping alone in a store, riding the bus, cleaning windows, or visiting a theatre. They felt happy and relaxed when they were able to do ordinary activities in their daily lives. Being able to do things by themselves provided a feeling of being free, which was said to be important for feeling well.

Well, I can feel … as an example … when I ski … then I feel … I haven't felt anything from my disease … instead, I have been able to stand on the slopes and feel the wind … and then you can feel that sense of freedom … yes … I can give it my all.

But everything is as usual … well, it does … when I'm mobile and normal … what I recognize as normal … and the over mobility is the opposite … You know I ran and called my mother and said to her that … mom, I have managed to run across the lawn.

### Being able to move on

When the women talked about feeling well, they said that it felt important to let go of their worries. Previous experiences were said to ease their ability to leave things as they are. The women described letting go of things that were not considered likely to change and things they had no control over. They talked about how they actively managed themselves in order to process a somewhat changed everyday life. Feeling well meant that they felt satisfied with life flowing on and steering away from gruelling work and exertion. There was an urge to be in the present moment, and simultaneously the women described themselves as being directed towards the future. Feeling well meant having confidence in oneself and one's ability to carry on in life calm and unruffled. The women with PD described that they felt well by letting go of their facade and being themselves. They took charge of their lives by focusing on choosing what they really wanted in life.

I feel calm now … I feel calmer and more secure … more self-confident … and I can … I know that I can do things despite of being ill … how should I put it … I can take place in life somehow … earlier you felt like you wanted to vanish … I claim space in society.

You feel well … and you feel that you are in a flow and that life is moving on and you feel satisfied with life … then you feel a [sense of] well-being.

### Feeling joy by being connected

Feeling well for women with PD means experiencing a feeling of belonging and connectedness with others. The women described that they felt needed and understood by friends who signalled that they cared. They felt supported by being engaged and taking part in various activities, for example, friends’ gatherings and choir practice, and they noted that it was important to feel accepted for who you are. The women also said that it was important to them that others around them felt well, and that family was an important source of feeling well and sharing joy. Being able to spend time together and to share experiences with family members, for example, grandchildren and children, was described as bringing happiness to their lives. The women with PD also talked about feeling well through professional development and by having employers who had faith in their efforts at work. Feeling well was said to imply having a place in the community and feeling valued.

It's [a] real delight with the children and you … they are so happy … when they stop by … he [grandson] is happy when we are together and he … you forget … and you get so much in exchange … you get so much love from the children … but you give them love as well … it is a delight.

Well … it feels good … how should I say… well … they [friends in the choir] accept me for who I am … and yes … they are so thoughtful, and if I don't show up, they phone me and ask me how I'm feeling.

### Finding peace and harmony

The women with PD described feeling well as finding mental and spiritual tranquillity. They talked about feeling well inside and of a well-being that was not connected to the illness or the ill body. There was a moving away from the burdensome body and forgetting about the illness by directing oneself towards the surroundings, for example, to engage in being in the here and now with others. Feeling peace of mind was possible, and they described that they could connect with their inner needs by meditating and through self-awareness. They felt calm just sitting down and relaxing, or viewing the colours when painting, taking a bath or a walk, and just doing things for their own sake. Feeling well also meant being in a creative mood and still experiencing a soothing environment. They could experience both calm and motion by being outside in nature, and sensing the fresh air made them feel free. They felt alert and happy during physical exercise and by being in motion.

The flowers and all the exuberance of colour and to make the plantings look like you intended … it's so beautiful to be able to get nice and tight plantings and the herbs, and [to] just swish your hands through it and just feel the fragrance … and footbath with peppermint … well, what should I say … it is all the things that give you pleasure and enjoyment.

Well, it is different sorts of things and it depends on what mood you're in, and I feel more and more of what's important for me … to be able to feel well I need to do the things that give me satisfaction … I need to get rid of the stress … and that becomes more and more important to me … there is a calmness in the colors and in what you are going to paint, and then you don't think of anything else than that.

### Being the director of one's own life

When feeling well, the women with PD described feeling less stressed and being able to turn down commitments. They said that it was important to give oneself permission to say no and by doing this, they also felt relief. Feeling well meant being in control and having the opportunity to choose how their days would be spent. It was important to have quiet surroundings and to take oneself into account to a greater extent than before becoming ill. Planning daily life was seen as promoting well-being, and having things in order and having foresight reduced stress when they needed peace and quiet. The women described planning moments of rest and sleep where their bodies could relax. Their prioritizing meant the possibility of living a good life, and planning was as important as being able to adopt an attitude of acceptance, when giving up daily plans was required due to a lack of strength.

I can feel in control … not in a bad way … instead, in a way that I feel that I am in control of the things that need to be controlled … I need control and foresight … no surprises … then I can relax and feel really well … and really satisfied.

I want a calm environment … I want … I learn to think before acting … I learn to say to others that I have to think about it [a commitment] before immediately just saying yes to everything.

## Discussion

As a result of this study, we suggest that meanings of feeling well as experienced by women with PD seem to encompass a stance where life feels pleasant and where it is possible to find repose in self-awareness and not feel estranged. Feeling well meant that they experienced their bodies as unnoticed; they felt that they were able to move on; they felt joy by being connected; and they felt peace and harmony. Gadamer (1996) has described how human health and well-being can be comprehended as silent. The character of our health can, therefore, be understood as concealed. It is not obviously presented to us, and we tend to take it for granted in our everyday living. From this study, we understand that for women with PD, being the director of one's own life can mean feeling well despite leading an everyday life influenced by bodily disruption and illness. In an existential understanding, well-being and illness are interwoven in human life. In this view, being human means living an everyday life faced with possibilities as well as limitations (Dahlberg, Todres, & Galvin, 2009).

For women with PD, feeling well from this point of view ought to be understood in relation to the fact of being ill and not as a separate phenomenon. Despite their illness, we understand that women with PD feel well when their illness is not the dominant experience in their everyday lives. Feeling well for women with PD can be understood as occurring when their bodies do not demand constant attention. This is similar to the results of Olsson et al. (2013) who showed that women with PD, who experienced fatigue, could enjoy days when they had strength to follow through with their daily living. These days felt easier to manage and by getting used to their experiences of fatigue, it felt easier for the women to keep up with interests and enjoy life together with friends and family. In this study, a less-strained and familiar body made it possible for the women with PD to move around easily with the body being in the background rather than in the foreground. The predictable body is described by Merleau-Ponty (1996) as simultaneously present and absent, providing the person unreflected attention to the surrounding world. It is through the body that the world becomes meaningful, and for women with PD, being able to do the ordinary resulted in feelings of being free. The notion of freedom can be understood as situated with an inner freedom independent from restraint (cf. Frankl, 1987). For women with PD, this can mean that feeling well is related to freedom despite living with illness.

The women with PD described how they were able to move on and to let go of things that they could not control or have an influence on. Kang and Ellis-Hill (2015) describe how people with PD used words such as fighting and facing up to being ill and the illness effects on daily living. In this study, we found that the women worked actively to process their changed everyday lives, and feeling well meant being self-confident and future-oriented. Dahlberg et al. (2009) advanced the Heideggerian concept of letting-be-ness and discussed how people who reconcile with the possibilities available can find well-being despite constraints. Despite living a daily life strongly constrained by illness, women with PD strive to not let the illness control them. Frankl (1987) described how a person's ability to influence his or her own approach to meeting the demands of life gives meaning. When women with PD described feeling well, they felt satisfied by steering away from gruelling tasks and exertion. By taking control and being the director of one's own life, they could feel well. According to Corbin (2003), it is possible to experience health when being able to recognize bodily information, and for women with PD, it can be understood that feeling well incorporates planning, self-awareness, and connecting with one's own needs. Relaxing and being in tranquillity were said to be a source of feeling well, and the women with PD pointed out that they considered their well-being to be related to being directed to their surroundings, and simultaneously as related to experiencing peace of mind.

Finding rest and stillness has been described by Dahlberg et al. (2009) as important for personal well-being by allowing oneself to just be. The women with PD narrated how they felt well by being creative and being in movement. They felt well by just sitting down and also by bodily exercise. Feeling well could imply gardening, sensing the fresh air, or simply taking a bath. Dahlberg and Segesten (2010) describe how human life is characterized by motion and movement. Health and well-being can be understood as containing both stillness and motion in people's everyday activities of life. For women with PD, feeling well can be understood as being in motion and also being still with an uninterrupted direction towards the surrounding world.

Women with PD narrated feeling needed and supported by others as a source of feeling well. There was joy in being connected and also in a sense of belonging with others. Studies by Juuso, Skär, Olsson, & Söderberg (2013) and Olsson, Skär, & Söderberg (2010) show that for women with long-term illnesses, feeling needed and understood can be viewed as vital in experiencing well-being. For women with PD, it was important to feel accepted for who they are and to be their own self. People's experiences of well-being are, according to Dahlberg and Segesten (2010), associated with identity and self-esteem. In a study on how people with PD live life successfully, Kang and Ellis-Hill (2015) stated that maintaining usual life and physical ability is to be seen as the main concern among people with PD. Kang and Ellis-Hill (2015) further describe how feeling accepted by others promoted a sense of normalcy among people with PD. Being yourself and living a desired life corresponds with the narrations of women with PD as they describe their striving to focus on their own inner needs, and also the importance of acceptance by others. Drawing on Heidegger's existential philosophy, Dahlberg and Segesten (2010) further discuss the human need for fellowship and being connected with others in one's surroundings. In this study, our understanding shows that, for women with PD, the stance where life feels pleasant and finding repose in self-awareness supports the women's experiences of well-being. The identification and support of health processes as a resource for a person living with illness is important when the goal is to support persons for an existence dominated by well-being and health (cf. Dahlberg & Segesten, 2010; Kang & Ellis-Hill, 2015; Toombs, 1993).

## Methodological considerations

In this study, we chose to interview women who were willing to describe their experiences about feeling well when living with PD. The interviews gave us access to both meaningful and varying expressions of the studied phenomenon from the women's point of view. Because the women were recruited from a patient association, there is a risk of bias in the selection process, that is, this sample selection encourages a certain type of informants that might have impact on transferability. Although, we view the rich descriptions that the women who participated in this study could provide us with as strengths related to theoretical richness and accuracy. The findings of this study can therefore be seen as applicable to the context of addressing the need to feel well among women who live with PD. At a general level, the findings can be applicable in the context of understanding and addressing the needs of persons who live with long-term illness. The results can be of interest not only in meeting women with PD, but also in meeting the needs and promoting capabilities of people who live with long-term illness in general in their strive for feeling well (cf. Gadamer, 1995). During the interviews, the women expressed their lived experiences freely, and our judgment is that they indicated that they understood the meaning of the questioned areas. The first author who conducted the interviews used probing interview questions, which strengthened the possibility of bridling the presupposed approach to the world in favour of not making definite what is indefinite, and instead questioning judgments of a certainty considered as general (cf. Dahlberg et al., 2008). This approach made it possible to let the women's points of view come forward. In using probing questions, the first author (MO) could control the interview in a phenomenon-focused manner. The use of probing questions also put forward the flexibility and spontaneity of the interviewer, and this encouraged sensitivity towards a phenomenological attitude. The first author also strived for being attentive so as not to end an area of questioning or an interview too early; this is an approach that, according to Dahlberg et al. (2008), is crucial for not leaving a phenomenological stance of objectivity. The interviewer's persistent questions and responses led the women towards reflection and expressing deepened layers of meaning (cf. Dahlberg et al., 2008). In the data analysis, we felt challenged by the women's rich descriptions and our understanding of a much complex phenomenon interwoven in the women's lives and the fact of living with a long-term illness. During the analysis, we have strived for a bridled approach related to figure and background concerning feeling well despite being ill and how this situation can be understood. According to Dahlberg et al. (2008), an essence is always understood against its horizons, an approach that we have worked with during the analysis when trying to understand the studied phenomenon.

## Conclusions

In this study, meanings of feeling well as experienced by women with PD seem to encompass a stance where life feels pleasant and where it is possible to find repose in self-awareness and not feeling estranged. Feeling well meant that the women with PD experienced their bodies as unnoticed; they felt that they were able to move on; they felt joy by being connected; and they felt peace and harmony. Meanings of feeling well imply a movement between being active and being in stillness. For women with PD, it is important to feel a belonging and a connectedness with others. Healthcare professionals can support women with PD in being active and engaged in various activities and gatherings, as this is stated as helpful in order to feel well. It was also of importance to find rest and to feel relaxed by being in motion or being connected to inner needs. If patient encounters with women who live with PD, the knowledge about their lived and experienced health processes can be used to understand and confirm their lived needs. It is therefore important to support women with PD towards balancing motion and stillness. From this, it is possible to support women with PD in finding relaxation and finding ways to interact and engage in daily living. This knowledge in the care meeting can support the women's striving to not let the illness be the dominant experience in their daily living.
